# Fostering social health of people with dementia: evaluation of *the Razem przed siebie* dementia awareness campaign in Poland

**DOI:** 10.3389/fpubh.2024.1418867

**Published:** 2024-08-21

**Authors:** M. Błaszkiewicz, D. Szcześniak, M. Ciułkowicz, Julia Ewa Rymaszewska, L.-F. Low, H. Brodaty, J. Rymaszewska

**Affiliations:** ^1^Department of Psychiatry, Wroclaw Medical University, Wrocław, Poland; ^2^Department and Clinic of Dermatology, Allergology and Venerology, Wrocław Medical University, Wrocław, Poland; ^3^Faculty of Medicine and Health, University of Sydney, Sydney, NSW, Australia; ^4^Centre for Healthy Brain Ageing, Discipline of Psychiatry and Mental Health, School of Clinical Medicine, University of New South Wales, Sydney, NSW, Australia; ^5^Department of Clinical Neuroscience, Wroclaw University of Science and Technology, Wrocław, Poland

**Keywords:** dementia, campaign, social health, evaluation research, stigma

## Abstract

**Background:**

Due to the need to increase social awareness about dementia and the needs of patients living with dementia in Poland, the *Razem przed siebie* (eng. *Forward with Dementia*) campaign was created. The aim of the study was to evaluate its effectiveness.

**Methods:**

To disseminate key campaign messages to the target audiences (people with dementia, carers, health and social care professionals [HSCP] and general public) a website, social and traditional media promotions, webinars and social activities were created. The campaign ran between September 2021 and April 2022. Mixed methods (online survey, reach estimates and interviews) were used to evaluate the campaign.

**Results:**

Almost 1,300 people visited the website during the campaign period. Of these, 55 carers and HSCP responded to the online survey. The most read section of the website was *Understanding the diagnosis* (carers [56% of 25] and HSCP [80% out of 30]). The website was mostly accessed by carers (68%) and HSCP (66.7%) through word-of-mouth recommendations. 80% carers and 90% HSCP found the website very or extremely helpful. Over 90% of carers and HSCP expressed an intention to revisit the website. Based on 31 interviews, campaign effects, change mechanisms and limitations were identified. Campaign events elicited positive emotions among people with dementia, providing them with a feeling of belonging and engagement. Esteeming personal interactions over informational campaign materials, those with dementia felt acknowledged and empowered by the events. Carers also reported positive experiences and increased interest and knowledge, though they expressed disappointment with the lack of respite care, an issue beyond the campaign’s scope. HSCP perceived the campaign events positively and identified significant gaps in the dementia care system.

**Conclusion:**

Evaluation of the *Razem przed siebie* campaign revealed successes and limitations. While effectively incorporating anti-stigma campaign recommendations and enhancing social health for individuals with dementia, the campaign clearly showed the pressing need for systemic solutions. Despite positive perception of the campaign, there is a need for a better diagnostic and post-diagnostic support for people with dementia and their carers.

## 1 Introduction

The WHO Global Dementia Action Plan 2017–2025 (1) recognizes social campaigning as a crucial means to raise awareness and friendliness about dementia. The recommended key messages of such actions are: to spread reliable knowledge about dementia, its subtypes, early symptoms and risk factors, as well as to counteract stigmatization and discrimination and to plead in favor of the human rights of people with dementia (1). In 2021, only 21% of WHO members had implemented dementia awareness campaigns (2).

Furthermore, reports from studies assessing the effectiveness of the dementia campaigns are sparse and not directly comparable due to different campaign goals, target groups, communication channels, societal contexts, and evaluation strategies (3–8). Available evaluation results of mass media campaigns on dementia from the Netherlands (4, 5), Belgium (5), and Australia (7) indicate only a partial change in the campaign goals, e.g., increased awareness of dementia risk factors among general public. The increase in knowledge was observed only in better educated demographic strata (5). Active participation by general public in the campaign events (not just exposure to promotional materials) allowed for better recognition of the campaign’s key messages (4). Australian *Forward with Dementia* (8) and Canadian *Not If, But When* (6) web-based resources aimed at health and social care professionals (HSCP) only partially influenced their attitudes regarding respectively: (1) diagnostic conversation for dementia and referral for post-diagnostic support, and (2) comfort in assessing driving risk in dementia, indicating that a social campaign is not a complete remedy for systemic barriers.

In Poland, according to estimates, there are currently over 500,000 people living with dementia, many of whom lack a formal diagnosis (9). Poland has yet to implement a national dementia strategy, and the health and social care systems function independently, complicating access to appropriate care (10). Informal carers, who often handle the coordination of treatment and care, have limited respite options (11). The few scientific studies on the social situation of people with dementia in Poland reveal that awareness of dementia is generally low among the general population as well as HSCP (12–14). Given the rising number of dementia cases, this lack of awareness constitutes a serious barrier to improving the situation for people with dementia in Poland (12). Despite calls from national advocacy organizations (15) and scientific reports (16) emphasizing the necessity of a nationwide social campaign addressing dementia, no such initiative had been undertaken until the commencement of this study. Prior sporadic health promotion initiatives related to dementia were typically confined to local efforts, and their efficacy remains unreported.

As meta-analyses show, there is no single recipe for campaign success (3). Their effectiveness is determined by a multitude of factors that require further research (3). Moreover, examination of many campaigns aimed at raising awareness and destigmatizing mental illnesses has shown that they often bring no benefits or, worse still, unintentional adverse effects (17, 18). For instance, concentrating on enhancing understanding of the biomedical aspects of a particular illness, while it diminished the tendency to blame patients for their condition, led to a rise in the perception that the disease is not amenable to therapy (18). Recognizing these failures allowed the researchers and activists to formulate guidelines to support effectiveness of public health campaigns (17–20). Advice included empowering individuals with firsthand experiences of mental health problems to spearhead grassroots social movements and to share their lived-experience; concentration on rights and dignity of those who have faced stigma and discrimination; substituting notions of incapacity and dangerousness with narratives of hope and competence. The context of the health care system and its limitations also needed to be addressed (17, 19). Interestingly, applying these indications to the context of dementia demonstrates their compatibility with the concept of social health (21). The paradigm of social health is one of the most prominent frameworks for explaining health for people with dementia, defining it as a dynamic process involving adaptation and coping with a chronic disease in social life (21, 22). Maintaining social health means balancing the deficits and limitations resulting from disease with personal and social resources, and environmental conditions (22). Social health depends on both how person with dementia interacts with the social environment and how the social milieu reciprocally interacts with them (21). A threat to this balance and reciprocity is societal stigma, which—within the social health concept—can be understood as depriving a person with dementia of: obligations, rights, and participation, i.e., essential elements of social health. Importantly, social health is regarded as a modifiable risk factor for cognitive decline (21), implicated in the pace of progression of dementia (23). It therefore appears that awareness campaigns may have the potential to enhance some aspects of social health in dementia (24).

The previously outlined strategies for impactful anti-stigma campaigns (18, 20) address changes at the individual level (as posited in the social health framework) (21). This involves empowering individuals in terms of their capabilities, social participation, and independence. The reinforcement of individuality, achieved through initiatives such as social campaigns, is intended to bring about transformations in the social environment, leading to, e.g., a reduction in stigma. In turn, decreased stigma may, like a feedback loop, bolster the individuality of those navigating the challenges of the disease.

Because the concept of social health is a priority in research on the health of people with dementia (21) and corresponds directly to recommendations for conducting anti-stigma campaigns (20), we utilize it as a conceptual framework for post-hoc analysis of the effects of introducing in Poland a dementia awareness campaign and website *Razem przed siebie* (in English-speaking countries, the campaign slogan was *Forward with dementia*) in the perception of dementia among the target groups. The aim of this study was to evaluate effects of social campaign *Razem przed siebie* in Poland using qualitative and quantitative methodological attitude including mapping campaign effects onto the social health conceptual framework.

## 2 Materials and methods

### 2.1 Intervention

*Razem przed siebie* campaign and website were part of an international Co-Designing Dementia Diagnosis & Post-diagnostic Care (COGNISANCE) project. An international consortium composed of five countries (Australia, Canada, United Kingdom, the Netherlands and Poland) developed the generic dementia awareness intervention. Based on close cooperation between researchers, a marketing company and local working groups composed of people with dementia, carers, HSCP and key stakeholders developed the branding and website design. The co-designing process and website user-testing have been described (25). The English name *Forward with dementia*, key messages and content of the website were translated and adapted to the Polish context. Through further cooperation between Polish research team, and local working group a campaign strategy was prepared. The leading team conducting the campaign consisted of four researchers and two volunteers from the Wroclaw Medical University, diverse in age, gender, educational background (psychiatrists, psychologists and medical students) and years of experience (both early career researchers and experienced independent researchers).

#### 2.1.1 Target audiences

There were four target audiences of the intervention in Poland: people with dementia, carers, HSCP and general public. Key messages were:

1. Dementia is a disease which needs to be diagnosed and treated.
2. Dementia diagnosis is the first step to starting appropriate therapy.
3. It is possible to live a positive life with dementia.
4. There are things that can be done to live well with dementia.

#### 2.1.2 Website

The campaign website was a resource about dementia for three groups of recipients: people with dementia, carers and HSCP. It contains articles on: the diagnosis process, acceptance of the diagnosis, coping with symptoms, living well with dementia, plans and decisions for the future, tailored in content to each recipient group. Additionally, the website presents personal stories of people with dementia and contained news and promotions on campaign events.

#### 2.1.3 Campaign

**Figure 1:**
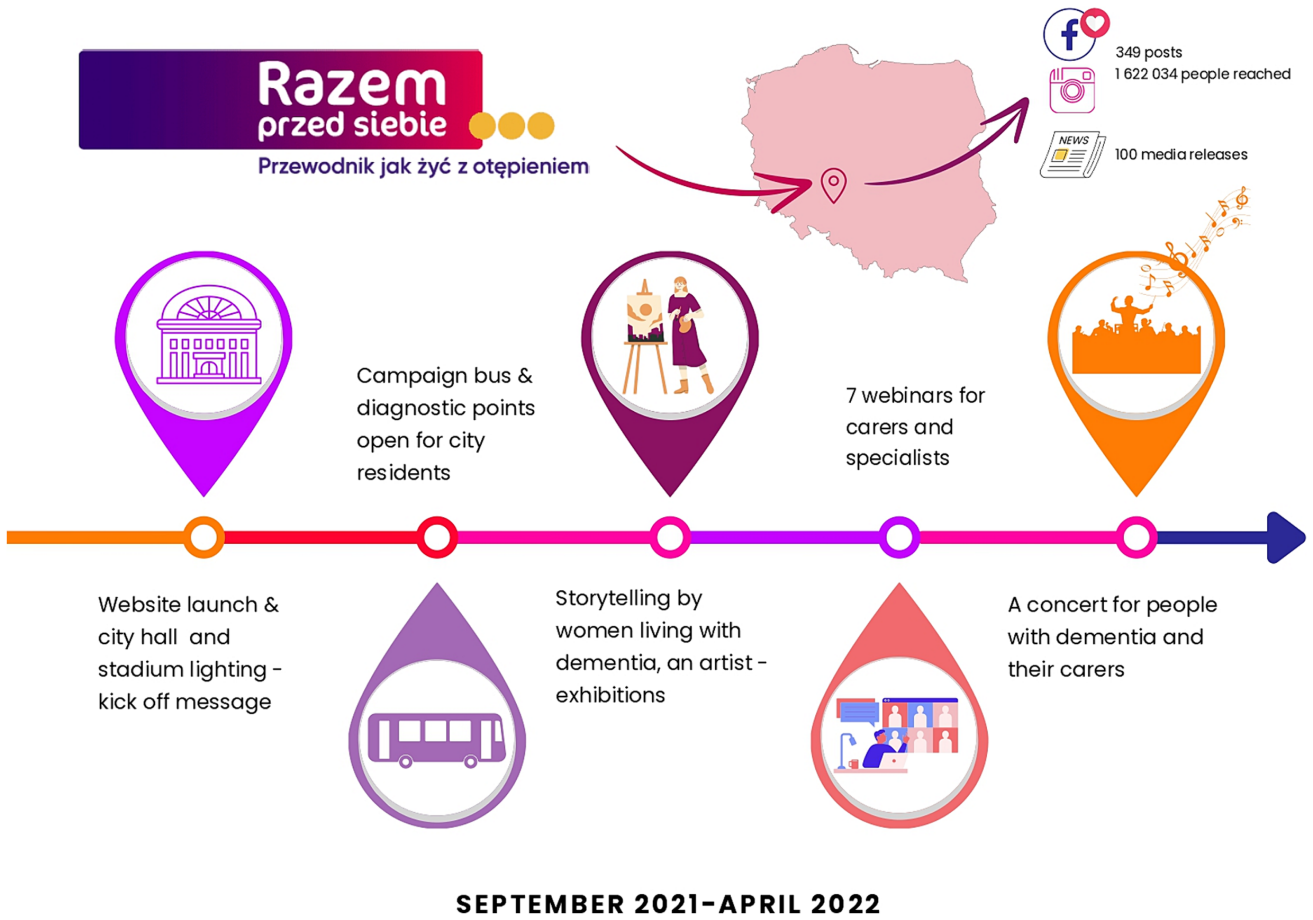
Highlights from the *Razem przed siebie* campaign and the Polish logo.

In Poland the campaign ran from 21st September 2021 to 7th April 2022. The main activities were concentrated in Wrocław and Lower Silesia. Key messages of the campaign were promoted via a range of educational and participatory activities (Figure 1) such as: social media marketing (Facebook, Instagram, YouTube), media coverage (local and nationwide press, radio and TV), distribution of printed leaflets, posters and gadgets (pens, badges, bags, reflective bands), promotional spots on public transport and regional railways, campaign bus covered with the campaign slogans, illumination of important buildings, webinars for carers and HSCP, exhibitions of paintings by a local artist living with dementia, speeches by campaign ambassadors (person with dementia and carer), mobile screening points, lectures in schools, senior councils, Universities of the Third Age, meeting centers for people with dementia, information stand at the seniors’ festival and final music concert. The campaign received honorary patronage from: the Polish Minister of Health; the Deputy Marshal of Lower Silesia; the Voivode of Lower Silesia; the President of the City of Wrocław; the city of Wrocław; the president of the Polish Alzheimer’s Foundation, Wrocław Women’s Council and the Rector of the Wrocław Medical University. A collection of photographs capturing various campaign events has been provided as the Supplementary material.

### 2.2 Intervention evaluation

#### 2.2.1 Design

A mixed-method approach was applied to assess the effects of introducing a dementia awareness intervention. Outcome measures were: website usability (operationalized by: time spent on the website, information read, website helpfulness, website information source, ease of use, plan to visit the website again and reach) and effects of the campaign among people with dementia, carers and HSCP. Quantitative design was used to evaluate the website usability among carers and HSCP and to estimate the reach of selected campaign activities. Qualitative study was aimed to assess the effects of the campaign among people with dementia, carers and HSCP. Study procedures were conducted in accordance with the Helsinki Declaration (26) and approved by the Ethics Committee of the Wroclaw Medical University (No. KB – 928/2021).

#### 2.2.2 Recruitment process and data collection

##### 2.2.2.1 Quantitative data

The website evaluation survey was conducted throughout the whole duration of the campaign. The survey was available on the *Razem przed siebie* website under the button *Rate the website* located on the top navigation bar. Incentives to participate in the study were also published on the *Razem przed siebie* social media channels.

Visitors to the website were invited to complete the questionnaire; after clicking *Rate the website* they were redirected to the online survey on the Survio^®^ platform. After entering the link to the survey, participants were informed that completion was equivalent to agreeing to participate in the study. The survey included multiple choice questions regarding the general opinion about the *Razem przed siebie* website, user experience, time spent on website and general demographic information, including participant’s role, i.e., family & friend of a person with dementia, HSCP, person with dementia or other. Additionally, each subsection of the questionnaire comprised of an optional segment where respondents had the opportunity to provide open-ended comments.

The reach of selected campaign activities was counted on the basis of: data from Google Analytics (website visitors), data from the marketing company (reach of the press release: online and printed publications); number of page views and likes (posts on social networking sites and webinars), counting participants of certain events (number of tickets for a concert, number of people examined at diagnostic mobile points), number of distributed printed materials (leaflets, posters).

##### 2.2.2.2 Qualitative data

For the qualitative part of the research convenience sampling strategy was used. Representatives from the campaign’s target groups, including people with dementia, carers, and HSCP, who attended the events were invited to take part in the research. During the events, researchers invited participants to take part in interviews and share their impressions of the campaign. Those who expressed interest in the study were contacted via phone or email, and interview dates were scheduled individually. Interviews with people with dementia were conducted at day meeting centers that organized outings to campaign events. Prior to interviews, the purpose was reiterated and informed consent was obtained from all participants involved. Interview guide included questions about general experiences with the *Razem przed siebie* initiative, brand perception, dissemination channels, personal impressions and feelings, relevance of key messages, impact of the campaign and website on knowledge, attitudes, and behaviors toward dementia. The interviews were conducted and audio-recorded by research team members experienced in qualitative data collection (MB, MC, JER). Demographic data were collected and stored in password-protected files.

#### 2.2.3 Data analysis

##### 2.2.3.1 Quantitative data

Descriptive statistics were presented as mean, standard deviation or counts and percentages. Calculations were made using the R package for Windows (version 4.3.2) (27).

##### 2.2.3.2 Qualitative data

Recorded interviews were transcribed into verbatim scripts by the research team members skilled in preparing materials for qualitative analyses. Before analysis, the transcripts underwent anonymization and proofreading. The review of transcripts for accuracy served as an initial step in the authors’ familiarization with the data. Four researchers—one psychologist, two psychiatry residents and one medical student—conducted data analysis. Thematic analysis, incorporating inductive and deductive approaches (28), was employed to analyze the transcripts in relation to the primary analytical question: what are the effects of introducing a dementia awareness campaign and website *Razem przed siebie* in the perception of dementia. During the initial phase, the most information-rich transcripts from interviews with people with dementia, carers and HSCP were independently analyzed by two researchers who generated initial codes answering the study questions through inductive analysis. Through discussion between the researchers, the codes were standardized and compiled into a codebook. Subsequently, the remaining material was analyzed by one researcher using deductive analysis based on the jointly-developed codebook. During the brainstorming session, two researchers (MB, DS) engaged in the previous stages of the analysis examined similarities and differences between the studied groups and clustered individual codes into themes and sub-themes. The relevance and naming of the formulated themes and sub-themes were discussed until a consensus was reached. Themes and subthemes were then analyzed post-hoc from the perspective of the social health concept and its specific markers. Through discussion between researchers (MB, DS), it was determined which of the previously formulated sub-themes aligned with markers of social health.

The interviews, transcription and analysis were carried out in Polish, while the results are presented in English. Each quoted excerpt was translated independently by two researchers. The translations were then compared and a discussed to ensure accurate conveyance of meaning between languages.

## 3 Results

### 3.1 Participants demographics

Fifty-five individuals, including family and friends of people with dementia and HSCP, responded to the online survey between November 2021 and June 2022 (Table 1). No people with dementia filled out the survey. Out of the 323 individuals who accessed the survey link, only 55 completed the survey, resulting in a completion rate of 17%.

**Table 1:** Surveys and interviews: the demographic information of the participants.

Surveys					
	N	Sex	Age (years)		
People with Dementia	0	*N* = 0	N/A		
Family & Friends	25 (45%)	Female *N* = 19 Male *N* = 5 Non-binary *N* = 1	Mean – 46 (SD = 18)		
HSCP	30 (55%)	Female *N* = 22 Male *N* = 8	Mean – 38 (SD = 16)		
Interviews					
				Living Arrangements	Time since the diagnosis of dementia (years)
People with Dementia	14	Female *N* = 8 Male *N* = 6	Mean – 79	Alone *N* = 6 With family *N* = 8	Mean – 2.5
Carers	9	Female *N* = 8 Male *N* = 1	Mean – 64	Alone *N* = 2 With family *N* = 7	Mean – 7
HSCP	8	Female *N* = 7 Male *N* = 1	Mean – 41	N/A	N/A

HSCP, Health and Social Care Professional; N, number; N/A, not applicable.

Thirty-one interviews were conducted between March and September 2022 with people with dementia [*N* = 14], carers [*N* = 9] and HSCP [*N* = 8] (Table 1). The time since dementia diagnosis in participants with dementia ranged from 1 month to 8 years. In the group of carers, the time since the diagnosis of their relative ranged from: 8 months to 20 years. Eight carers stated that they provide the main care for a person with dementia. The group of carers included: spouse [*N* = 3], sibling [*N* = 1], children [*N* = 4] and grandchild [*N* = 1]. Six HSCP were healthcare providers and two social care providers. All interviewees were Poles, living in Wroclaw, Poland.

### 3.2 Quantitative data

#### 3.2.1 Survey

**Figure 2:**
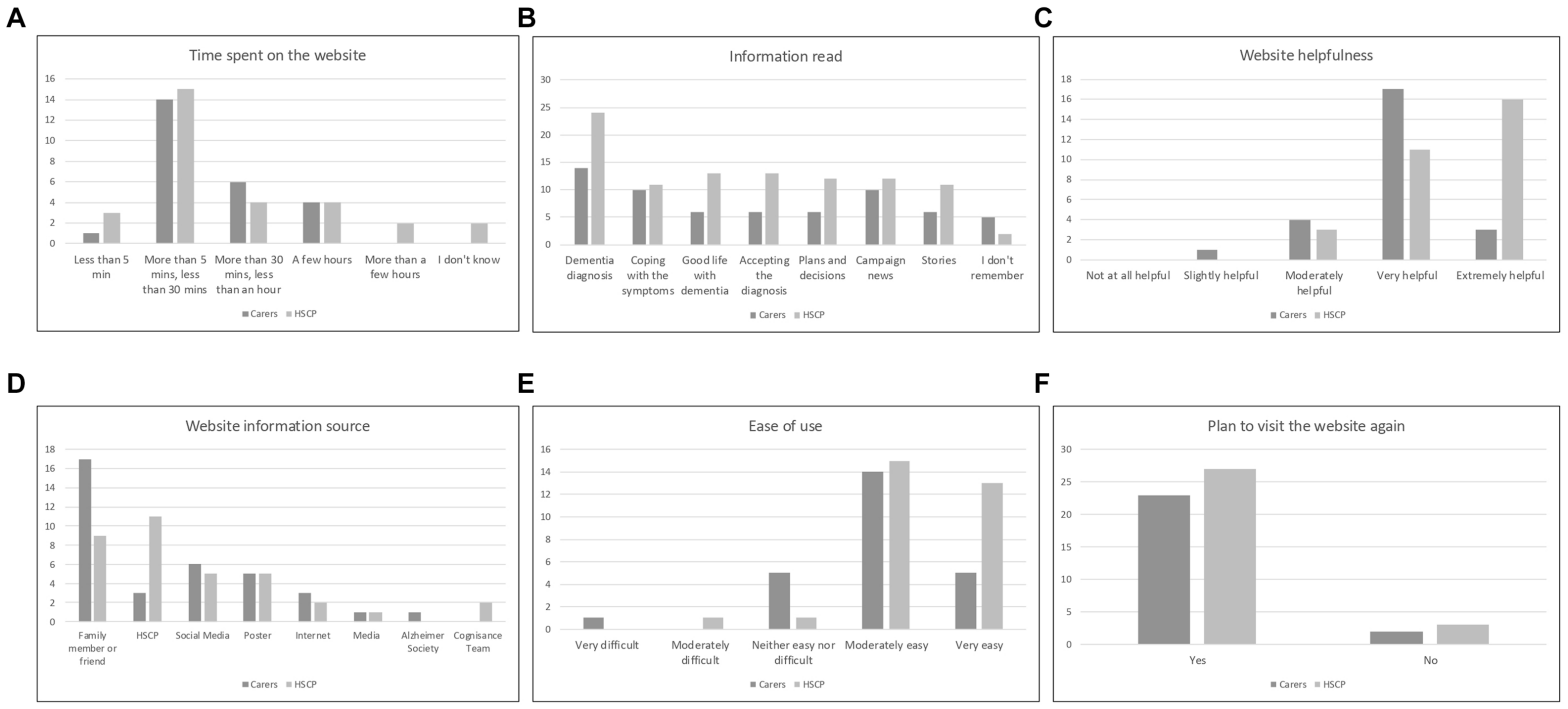
Survey data. **(A)** Time spent on the website; **(B)** Information read; **(C)** Website helpfulness; **(D)** Website information source; **(E)** Ease of use; **(F)** Plan to visit the website again.

The average time spent on the website (Figure 2A), both for carers and HSCP, was most often above 5 min and below 30 min. Only one carer and three HSCP spent less than 5 min on the website. The rest of the participants declared that they spent more than 30 min on the website.

The most read section of the page (Figure 2B) was *Dementia diagnosis*, both for carers (56%) and HSCP (80%). Carers also showed interest in the *Coping with the symptoms* section and *Campaign news* (40% each), and—to a lesser extent—in the *Good life with dementia*, *Accepting the diagnosis*, *Plans and decisions* and *Stories* (24% each). The HSCP expressed approximately equal interest in all the remaining sections.

The majority of carers (68%) found the website very helpful (Figure 2C). 12% claimed the website was extremely helpful; for 16% it was moderately helpful. One carer answered the website was only slightly helpful. Most of HSCP rate the website as extremely helpful (53.3%) or very helpful (36.7%). For 10% of HSCP the website was moderately helpful. None of the participants acknowledged that the website was entirely unhelpful.

The most common source of information about the website (Figure 2D) for carers was family and friends (68%), for HSCP—their colleagues (i.e., other professionals—36.7%, and family and friends—30%). Only 12% of carers learned about the website from HSCP. For carers, the next most frequent sources of information were: social media, posters, Internet, and—to a lesser extent—traditional media and Alzheimer’s society. For HSCP, other common sources of information were: social media and posters, Internet and Cognisance Team members.

56% of carers claimed that the website is moderately easy to use (Figure 2E) and for further 20% it was even very easy. Also 20% agreed that the website is neither easy nor difficult to use. Only one carer found the website very difficult to use. For majority of HSCP the website was moderately (50%) or very easy (43.3%) to use. The vast majority of the visitors expressed an intention to revisit the website (over 90% for both carers and HSCP; Figure 2F). Less than 10% of all participants indicated that they would not visit the website again.

#### 3.2.2 Reach

The reach of selected activities, those which could be quantified, are presented in Table 2. The values provided apply only to the campaign period. Many participants might have had numerous interactions with campaign messages via various channels. Due to the wide array of events encompassed within the campaign and its promotion through numerous partners, we could not estimate the reach of all promotional activities.

**Table 2:** The reach of selected campaign activities.

Activity	Reach (at the end of the campaign)
Website visitors	1,282 visitors
Media release (91 internet publications, 8 printed publications, 1 TV coverage)	reach 1,503,000, 00
Distributed printed materials	900 leaflets 100 posters 1,150 gadgets
Social media channels Facebook Instagram	reach 1,622,034, 00 387 likes 61 followers
Webinars (on YouTube channel)	873 views (the most popular webinar on social health watched by 273 people)
Five mobile screening points	300 people examined
Music final concert	350 participants

### 3.3 Qualitative data

**Figure 3:**
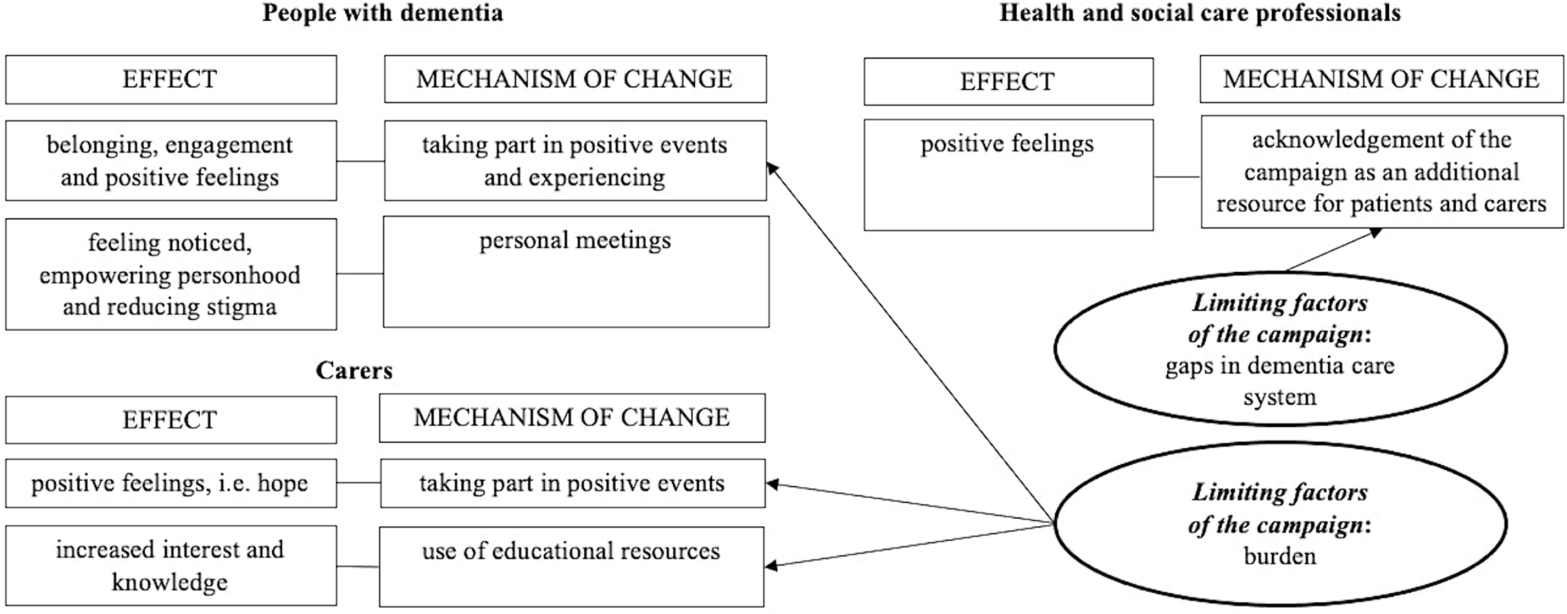
Qualitative themes arrangement.

The thematic analysis resulted in the identification of: (1) campaign effects and (2) underlying change mechanisms, individually for each of the studied groups. Additionally, factors that were external to the campaign, but influenced its effectiveness were distinguished. Arrangement of these themes is illustrated in Figure 3. The most representative quotes are presented in the text.

#### 3.3.1 People with dementia

***Mechanism of change: taking part in positive events and experiencing.***

***Effect: belonging, engagement and positive feelings.***

The campaign offered many events in which people with dementia took part. These events were often not directly informational, but concerned joint activities, interactions with others or artistic experiences. People with dementia recalled these experiences with enthusiasm and attributed positive emotional meaning to them.

*“I did not always get the idea but I had a feeling that it* [the campaign] *was something important.”* [Female, Person with dementia, 80].

*“I do not remember everything, but it was very interesting. And I was happy to go to such a meeting, because I’m always curious to hear something new.”* [Female, Person with dementia, 75].

*“We had a great trip! We saw exposition of the painter living with dementia”* [Person with dementia, Female, 79].

Campaign events enabled people with dementia to become active and meaningful participants in the community. Through joint activities and interactions with other people, especially those sharing similar experiences, they received a sense of social support and belonging to a larger social group.

*“I have found out that there are many, many of us, yes, who want to hear something, learn something. (…) I thought to myself that it would be good to train the mind a little, train myself and to do this being among other people. To be able to function, to cope.”* [Person with dementia, Female, 79].

***Mechanism of change: personal meetings.***

***Effect: feeling noticed, empowering personhood and reducing stigma.***

People with dementia emphasized that the campaign’s information materials, such as leaflets, posters or the website, had almost no effect on them. Cognitive difficulties and technological barriers prevented people with dementia from using written campaign resources, including the website. However, the most effective communication channel for them was personal meetings with other people who spread the campaign’s key messages.

*“To be honest, I do not read any leaflets. I just listen to the people [at daycare facility], what they say and suggest”* [Person with dementia, Female, 79].

*“It matters what someone says to me. When I read, I forget it right away”* [Person with dementia, Female, 79].

*“Well, you would have to, you would have to have someone else next to you who would bring these brochures and talk to you.”* [Person with dementia, Female, 80].

*“I am sorry, I do not use internet. I do not even have it. My granddaughter does.”* [Person with dementia, Male, 83].

The campaign made people with dementia feel noticed, supported and understood. Publicizing the topic of dementia gave them the feeling that they were not left out. People with dementia were also pleased that the campaign was being led by younger generation. The personal involvement of the team organizing the campaign in contacts with its recipients inspired trust in the initiative.

*“I am glad we have you. I feel calmer that you are dealing with this topic”* [Person with dementia, Female, 79].

*“Your project is for people like me. So that other people will start to look at us differently, so that they will not laugh, so that they will understand.”* [Person with dementia, Female, 83].

*“I wasn’t always able to get the point, understand it all. This whole initiative. But I always thought it was smart. And you, young people ... those people who sit there* [participants of the day care facility] *need you, consciously or unconsciously. Because I do not know how they perceive it. It’s not always possible to do something with our state, but we know we have help.”* [Person with dementia, Female, 80].

#### 3.3.2 Carers

***Mechanism of change: taking part in positive events.***

***Effect: positive feelings,* i.e.*, hope.***

Carers took part in campaign events, usually accompanying people with dementia. Carers appreciated the positive emotional influence of these initiatives and were personally connected. The character and tone of the events resonated with their feelings and personal experiences.

*“We were at an interesting conference where we heard about other events. It gave a lot of hope.”* [Carer, female, 55].

*“Definitely, beautiful activities were prepared. We talk to artist* [campaign ambassador – painter with dementia]. *An amazing person! I admire her very much.”* [Carer, female, 62].

Carers rejoiced in the positive effect that taking part in the campaign events had on the people with dementia. They also observed the impact of these positive experiences on their own emotional well-being and motivation to act. Carers emphasized that participating in the campaign gave them hope that living with dementia is possible and can still be valuable.

*“For the next two weeks, my mother was fascinated and often mentioned her experiences from the concert.”* [Carer, female, 62].

*“Sometimes I get burned out with care. Then you need a few days of rest. The events of the project motivated me to continue the care.”* [Carer, female, 62].

*“When you are a carer, you catch everything regarding the topic* [dementia] *that provide you with any hope for better health and life.”* [Carer, female, 55].

***Mechanism of change: use of educational resources.***

***Effect: increased interest and knowledge.***

The carers expressed a strong interest in the informational materials and content on the website. They declared that they had thoroughly reviewed the information resources. The fact that the campaign was developed by scientists from medical university was valued. Carers wished that the website would be expanded, and the materials would be more widespread and accessible.

*“The webinar was genuinely interesting. It had an accessible, popular science form. I think it would be of interest even to people who are not struggling with this problem. You can listen to it again on YouTube and you do not need to be there.”* [Carer, female, 28].

*“I am interested in new materials on the site. I regularly look there to see if there is something new.”* [Carer, female, 62].

*“Those who were not interested might have problems with noticing the campaign. I was hoping that there would be more advertising materials, for example posters.”* [Carer, female, 50].

*“Yes, I would recommend the website to others. I believe in competent people. And I consider you and your initiative as such.”* [Carer, female, 61].

Carers pointed out the importance of acquiring knowledge about dementia, particularly in managing its symptoms, fostering empathetic understanding of the sick person, and preparing for the future. They observed an increase in their knowledge due to the campaign, perceiving it as a response to the existing gaps in the resources for carers.

*“Such a project is very necessary, because you hear that more and more people have dementia and the people who care for them have problems of* var*ious kinds. You have to know how to deal with a sick person and how not to get angry with them… It opened my eyes a lot. There will be more to come, probably, more things that are incomprehensible and tiring and that this disease will simply not retreat, that it will not get better, just the opposite.”* [Carer, female, 75].

*“I was very pleased when I heard about the project. A year earlier, I sought help in many places, but there was very little information.”* [Carer, female, 62].

*“At first, we got angry or laughed at grandma. We could not cope. I started to understand what dementia is thank to the campaign.”* [Carer, female, 28].

***Limiting factors of the campaign: carer time and burden.***

Carers pointed out also the limitations of the campaign’s effectiveness. They referred to the dependence of a person with dementia on their support, their workload and struggling to reconcile time for caring responsibilities and work. As a consequence, the carer’s time constraints did not allow them to attend campaign events with the person with dementia. Moreover, caregivers pointed to gaps in the care system and lack of respite care, which the campaign cannot address. The campaign key message about hope for a positive life with dementia was perceived by some as overpromising and, in the face of the shortcomings in the care system, disappointing.

*“When I was at work, no one could give my mother a lift to an event.”* [Carer, female, 62].

*“What would I add to the website content? More information on the forms of institutional assistance. I know how it looks like in Poland. There is little support. Perhaps to website could present incentives to create such places* [support centres for PwD]*.”* [Carer, female, 61].

*“Materials are interesting, and they cared a lot to use words that give hope that you can still live your life. On the other hand, reality hits hard and one could be disappointed.”* [Carer, female, 55].

#### 3.3.3 HSCP

***Mechanism of change: acknowledgement of the campaign as an additional resource for patients and carers.***

***Effects: positive feelings.***

Professionals found the campaign valuable mainly as an information source for caregivers of their patients. They were pleased that such an initiative existed, enabling them to recommend it to individuals dealing with dementia and their caregivers. However, they perceived limited direct impact on enhancing their skills and practices.

*“I got acquainted with the website recently. I do not use it to develop my professional skills. But I can offer it as a reliable resource for my patients.”* [HSCP, female, 30].

*“Very nice, clearly made. That the people who are struggling with this problem could certainly find something for themselves.”* [HSCP, female, 50].

*“I flicked through section for professionals mostly but I strongly recommended news and stories for my patients. I gave them the website’s address on the piece of paper so they can search for it.”* [HSCP, male, 27].

*“I recommend the website whenever I see the need in my everyday practice. Especially to the carers, because most of patients with dementia are not able to use the internet.”* [HSCP, female, 51].

HSCP experienced positive emotions in response to the campaign and website launch. The campaign events were perceived as filled with hope and positivity. Raising the topic of dementia through the *Razem przed siebie* initiative in public sphere was assessed as very needed and valuable.

*“There has been a break in dementia psychoeducation in the last two years* [pandemic period], *so I enjoyed the campaign all the more.”* [HSCP, female, 55].

*“I enjoyed the opening conference as it was interesting fulfilled me with hope.”* [HSCP, female, 55].

*“I loved the concert! It would be great to repeat such event and make campaign more visible.”* [HSCP, female, 50].

***Limiting factors of the campaign: gaps in dementia care system.***

HSCP referred to the reality of people with dementia and their caregivers in the Polish care system. They emphasized that the campaign was unable to address the shortcomings in dementia care, i.e., difficulties in obtaining a professional diagnosis, insufficient number of places in care facilities or lack of respite care for carers. Campaign key messages and images may even be overly optimistic, fostering hope that might be broken when confronted with realities.

*“It all looks so perfect on this website that actually everyone is hugging, and life is good, and in practice, as I observe it, it is very different. Of course, it’s not, as I say, it’s not like a death sentence right away and something terrible. And in fact, there are a lot of seniors who really enjoy life, despite the diagnosis and the dementia. And they are really great people. However, this is also not so colorful and joyful always. I think that campaigns often show it like that, and, and this ... is not such a black and white image.”* [HSCP, female, 24].

*“I did not find a tab with specific addresses of institutions in different regions. Caregivers are not so much willing to read as caring looks like. They need specific information on what they can do, where to go.”* [HSCP, female, 30].

*“There is too little institutional help. These outposts are overcrowded. Cafes for seniors could also be created. As a result, caregivers are overburdened.”* [HSCP, female, 56].

*“We have a lot of specialists in a large city, but it is important that people in small cities are aware of where they can go.”* [HSCP, female, 55].

#### 3.3.4 Mapping campaign effects onto the social health conceptual framework

Post-hoc analysis of the qualitative results allowed for mapping the effects of the *Razem przed siebie* initiative onto the social health framework (Table 3) (21). This analysis reveals its empowerment effects on some of the individual and social environment markers of social health among people with dementia. Taking part in positive events of the campaign and experiencing them reinforced the social participation among individuals with dementia and reaffirmed the idea that they retain their social capabilities, despite the disease. The presence of ambassadors with first-hand experience of dementia signaled that people with dementia have social rights and can still fulfill social obligations and specific roles (e.g., an artist or wife). Contact with others during the campaign also influenced the social environment of people with dementia by strengthening its structure, reciprocity, and enabling for its more positive appraisal. As a result of the campaign, carers were able to shift their attention toward the more positive facets of their caregiving responsibilities and acquire knowledge that enhanced their ability to support the social health of people with dementia. In turn, HSCP acquired supplementary psychoeducational resources which they can distribute to their patients to offer additional support following diagnosis. However, alterations at the social environment level were restricted and not comprehensive. The campaign did not instigate notable alterations in the structure of the social environment in terms of facilitating access to post-diagnostic care, introducing long-lasting solutions in care system, or providing respite options for carers. In the context of stigma, the campaign addressed only some of its aspects. It did not affect its structural dimension.

**Table 3:** Mapping *Razem przed siebie* campaign effects onto the social health conceptual framework.

	Level	Domain	Markers examples
The concept of social health	**INDIVIDUAL**	**Capacities**	***Reciprocity***
		Independence	*Autonomy*
		**Social participation**	***Social Participation, Social Engagement, Social Leisure, Activities, Social Isolation***
	**SOCIAL ENVIRONMENT**	**Structure**	***Frequency of Contact, Social Network, Living Alone, Martial Status***
		Function	*Inability to help, Exchanging support*
		**Appraisal**	***Loneliness***

Bold font indicates areas implemented in the *Razem przed siebie* campaign. Adapted from: Vernooij-Dassen et al. (21).

## 4 Discussion

Our research contributes to the limited body of evaluation studies on dementia campaigns by expanding it to a novel cultural setting. To our knowledge, this is the first study evaluating a dementia campaign conducted in Poland. The evaluation outcomes suggest that the campaign and the associated website were very positively received. It effectively impacted several principles of the social health framework. Moreover, the campaign made strides in addressing the stigma associated with dementia, fostering a greater understanding of the social health dimensions essential to improving the well-being of individuals with dementia. Campaign effects, underlying mechanisms of change as well as significant limitations influencing the effectiveness will be discussed.

An integral element of the *Razem przed siebie* initiative was a website that condensed the educational message and disseminated news about the ongoing campaign. The website received very positive feedback across various dimensions from both carers and HSCP, highlighting the widely noticed demand for web-based knowledge resources (29). The majority of visitors commended the user-friendly interface and reported that the content captivated their attention for a considerable duration, prompting them to visit the website again. The most users visited the *Dementia diagnosis* section, potentially influenced by its positioning as the first thematic section. Nevertheless, this trend could also suggest that the key messages of the campaign and website held significant relevance at the onset of the dementia journey. Further, the predominant means of learning about the website was through word-of-mouth recommendations, originating from friends, family, or colleagues. As research indicates (30), this mode of information dissemination is common in healthcare and also in our study turned out to be effective. Therefore, it seems that the lesson for the future is not to rely solely on websites that passively convey health messages to people, but that more active, grassroots campaigns are required with different types of activities that people can engage with and talk about. Disappointingly, very few carers learned about the site from HSCP. However, this pattern should be evaluated over the long term, as during the study period, the promotion of the website among HSCP was still in progress.

Importantly, no person with dementia completed the website evaluation survey. A similar problem was noted by researchers from Australia (8), who struggled to recruit people with dementia to evaluate their campaign, even though, unlike Poland, they have programs supporting patient involvement in research (31). Absence of respondents diagnosed with dementia suggests that despite careful preparations (25), the website may not have been adequately tailored to the needs of people living with dementia (e.g., outlined in the DEEP Guide) (32). Furthermore, it underscores the prevalent challenges faced by older individuals in accessing digital resources, stemming from limited digital literacy and technological proficiency (33). This observation hints at the potential ineffectiveness of Internet sources, particularly conventional ones, targeted toward individuals with dementia.

The conclusions regarding the limited usability of the *Razem przed siebie* website for people with dementia are supported by the findings of our qualitative research. In our study, individuals with dementia indicated a deficiency in Internet usage skills and found written materials from the campaign less useful due to difficulty in assimilating new information. In spite of that, their perception of the campaign was mainly shaped by the emotional experiences they had during the events they attended (such as excitement, joy, pleasure, etc.), as well as the positive appraisal of the contacts with other people (feeling of connection with others). This finding aligns with evidences that emotional cues enhance memory in people with dementia (34), and emphasizes the potential of emotional communication as a foundation for dementia-friendly initiatives (34, 35). Moreover, it indicates that the design of *Razem przed siebie* campaign adhered to recommendations for creating initiatives aimed at mitigating the social exclusion of people with dementia (36, 37), primarily by offering them opportunities for meaningful engagement within a stimulating community environment (36, 38). Interviewees also acknowledged the significance of meetings with campaign ambassadors, which highlights the importance of empowering individuals with firsthand experience of dementia during social campaigns (17, 18, 20). Interestingly, some study participants emphasized the importance of involving young people in the campaign, indicating substantial potential for integration initiatives and multi-generational projects in dementia (36, 39). By mapping the results of our analysis to the social health framework, it can be inferred that the campaign contributed to bolstering the social health of people with dementia, both on an individual and societal level (see Table 3). Thus, contributing to overcoming social stigmatization.

The effectiveness of the campaign among individuals with dementia heavily relied on the capabilities of carers. Commonly, carers were responsible for seeking information about the campaign and accompanying individuals with dementia to the events, offering transportation and necessary support. Carer burden, frequently reported among this group in Poland (15) and globally (40), influenced whether a person with dementia could benefit from the event. This highlights that the campaign did not offer carers a form of respite from caregiving responsibilities, which is often sought in psychosocial interventions in dementia (36). Despite this notable barrier to the campaign’s effectiveness, carers also found *Razem przed siebie* to have a positive impact on their caregiving role. Participating in positively charged events allowed caregivers to witness the enjoyment of their loved ones with dementia, share moments of fun together, and experience positive emotions themselves. This translates into a tangible reinforcement of the positive aspects of caregiving, including sense of competence in providing care, strengthening relationships with a person with dementia, and fostering hope for the future in the journey with dementia. This is particularly important in the light of research results indicating that strengthening the positive aspects of care is necessary to maintain a good quality of life for carers and to protect them from adverse effects of caregiving (41). Unlike people with dementia, carers extensively utilized the educational resources provided by the campaign and benefited from the digital materials (which, especially since the pandemic, have proven to be convenient and time-efficient medium for Polish carers (42)). The need for access to reliable information about the disease highlighted by this study is consistent with the results of other research on the well-being of carers indicating that knowledge of dementia is a basic mean to better undertake the caregiving role and to prevent and manage specific situations (41, 43). Crucially, carers we surveyed highlighted that the information acquired during the campaign broadened their biomedical knowledge about dementia. Most importantly, it also enhanced their understanding of behaviors and approaches to communication with people with dementia. This outcome indicates that the *Razem przed siebie* campaign promoted the principles of person-centered care (44, 45), acknowledged as the highest standard of care for people with dementia (46, 47).

It should be noted, however, that in our study a few carers, along with some of the HSCP raised concerns that while the campaign’s key messages may instill hope, they could ultimately prove disappointing when confronted with reality. Consequently, the *Razem przed siebie* campaign may have echoed the trend identified in the literature on the cultural images of dementia (48), wherein there is a tendency to portray life with dementia in an excessively optimistic manner. As indicated by research (49), these overly positive images could result in unintended consequences, such as worsening stigma by not adequately portraying the challenges faced by individuals with dementia, potentially causing those who are not living well to feel like they had failed. Providing positive information about diagnosis (that the campaign encouraged) is insufficient, if there is no benefit through supports and services. Both carers and HSCP highlighted that the campaign materials failed to provide specific information about post-diagnostic care facilities and voiced discontent over the lack of systemic solutions for post-diagnostic support in Poland. Therefore, the campaign failed to address the needs of carers and HSCP in terms of enhancing their perception of availability of post-diagnostic support, a need that is not only important in Poland (15), but also elsewhere (50). Werner et al. reported that the perception of a lack of institutional support (conceptualized as structural stigma) that is associated with an increase in caregiver burden (51). Further, it seems the campaign did not prompt significant changes in the structure of the social environment (interpreted as a dimension of social health) for people living with dementia.

The surveyed HSCP, apart from referring to systemic barriers limiting the impact of the *Razem przed siebie* initiative, expressed their satisfaction that a new, reliable source of knowledge about dementia had been created. Nevertheless, professionals viewed the campaign and the website almost exclusively as resources they could recommend to their patients. Like the web-based initiatives in Australia (8) and Canada (6), the *Razem przed siebie* in Poland had limited effect on altering the professional practices of the surveyed HSCP; this can be understood in different ways. Firstly, the campaign design might have been overly ambitious concerning the intended target groups. With limited resources, a small team, and only local reach, it struggled to develop precisely targeted key messages and tailored promotional strategies. Alternatively, as in the Australian and Canadian cases (6, 8), HSCP may perceive significant systemic shortcomings that make them feel unable to offer proper post-diagnostic support to their patients. They may also lack hope that changes in their approach to dementia could improve the situation for people with dementia and their family carers. This aligns with the common “nothing can be done” mindset seen among HSCP, reflecting therapeutic nihilism, feeling of hopelessness, and a perceived lack of agency in managing dementia in their patients (52–54).

While the research results presented offer multiple perspectives regarding the effectiveness of the *Razem przed siebie* campaign in Poland, its limitations should also be considered. The study’s design relied solely on a post-campaign evaluation. Although formative research was conducted prior to the development of the campaign, it cannot be used to directly compare or evaluate behavioral changes resulting from the campaign. Moreover, the study’s timeframe did not allow for testing potential long-term outcomes of the campaign and the website use. Additionally, the qualitative approach used in the study has inherent limitations. While it provides in-depth data on the audience’s personal experiences, it restricts the representativeness of the findings (55). In turn, in the context of website evaluation, relying solely on a self-report questionnaire may not accurately capture user behavior. Therefore, it is advisable to supplement these data with more comprehensive insights obtained from online tools for analyzing website statistics (56, 57). Another challenge was participation bias (58). The study included individuals who proactively sought dementia information or had received support from dementia-related institutions. They were enthusiastic about and willing to participate in campaign events, as well as interviews or surveys. Moreover, due to the extensive distribution of the campaign across numerous channels, we encountered challenges in estimating the full scope of audiences and surveying a representative sample of them. Since, to our knowledge, this is the first evaluation study of a dementia campaign conducted in Poland, it is not possible to compare the current results with outcomes of other initiatives conducted in similar cultural setting. Our findings provide a reference point for stakeholders and researchers interested in developing and evaluating future dementia-friendly initiatives.

## 5 Conclusion

In conclusion, the analysis of the results presented enables us to identify areas where the Polish *Razem przed siebie* campaign proved effective and where it fell short. Firstly, the campaign successfully incorporated specific recommendations for effective anti-stigma campaigns and, moreover, these efforts were noticed and appreciated by target audiences. In terms of social health, on an individual level, involvement in the campaign facilitated the enhancement of social participation among people with dementia and reinforced the notion that they maintain their social capabilities, despite the disease. The campaign can also be viewed as a catalyst for change within the social environment of people with dementia. Thanks to the campaign, carers could focus on more positive aspects of their caregiving role and gain knowledge enabling them to better support the social health of a person with dementia. In turn, HSCP obtained additional psychoeducational resources that they can share with their patients to support them after diagnosis. However, the very positive reception of the campaign by the majority of respondents should not be taken for granted. The inclination toward such favorable evaluations may stem from a dearth of alternative sources and may operate under the principle of *something is better than nothing*. The shortcomings of the campaign must also be acknowledged. The campaign’s tone was sometimes perceived as overpromising and not entirely tailored to the realities of the Polish health and social care systems, which may trigger unintended additional frustration and disappointment. Also, the assumptions regarding effectively reaching such diverse target audiences proved to be overly ambitious for a campaign with limited organizational resources. The campaign’s key messages had the least influence on HSCP, indicating a need to explore alternative initiatives targeted at this group.

It is evident that the campaign formula was also unable to overcome barriers arising from the existing social welfare system, e.g., alleviating carer burden. The *Razem przed siebie*, albeit local in scope, may attract the attention of local and state authorities to the plight of individuals with dementia and underscore the necessity to establish the long-term systemic solutions that addresses the needs of people with dementia and their carers. Considering the presented findings, it is urgent to develop and implement nationwide, evidence-based social campaigns aimed at impacting the social environment of people living with dementia. Integrating awareness initiatives into dementia health policy should be a permanent fixture. This study may also encourage further research evaluating dementia campaigns, as the insights gleaned from such evaluations facilitate the development of subsequent, more tailored and effective interventions. Equally crucial is the necessity to document unintended consequences of the campaigns, serving as a cautionary note for the creators of future actions.

## Data Availability

The raw data supporting the conclusions of this article will be made available by the authors, without undue reservation.
