# Supplementary material for: Fostering social health of people with dementia: evaluation of the Razem przed siebie dementia awareness campaign in Poland
Source: Front Public Health. 2024 Aug 21;12:1418867. doi: 10.3389/fpubh.2024.1418867 (PMC11371569; doi:10.3389/fpubh.2024.1418867)
Supplement: Supplementary file 1 [file Data_Sheet_1.PDF]

Supplementary Material

Highlights from the *Razem przed siebie* campaign

Launch of the *Razem przed siebie* campaign – 21st September 2021

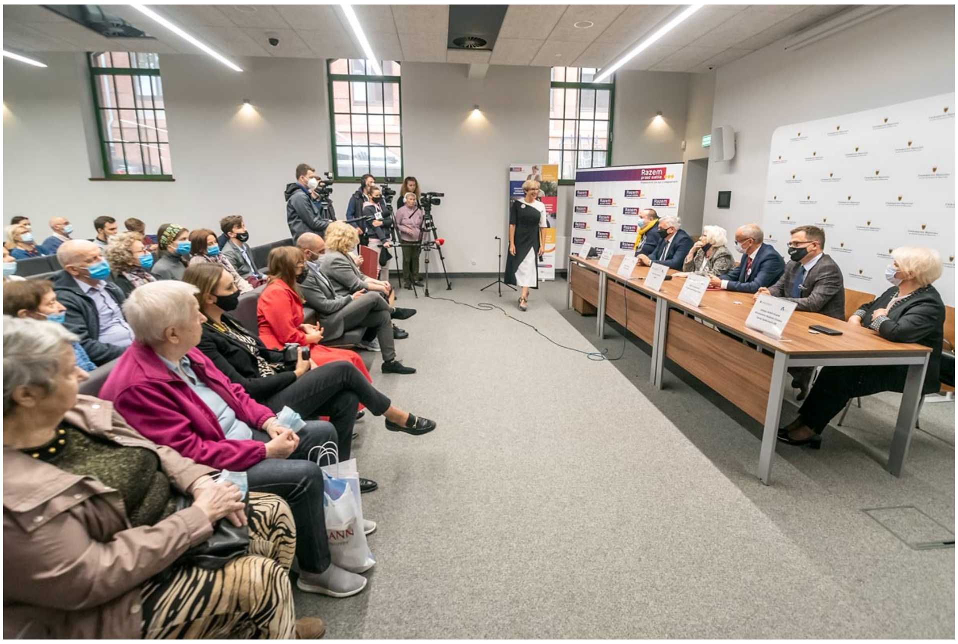

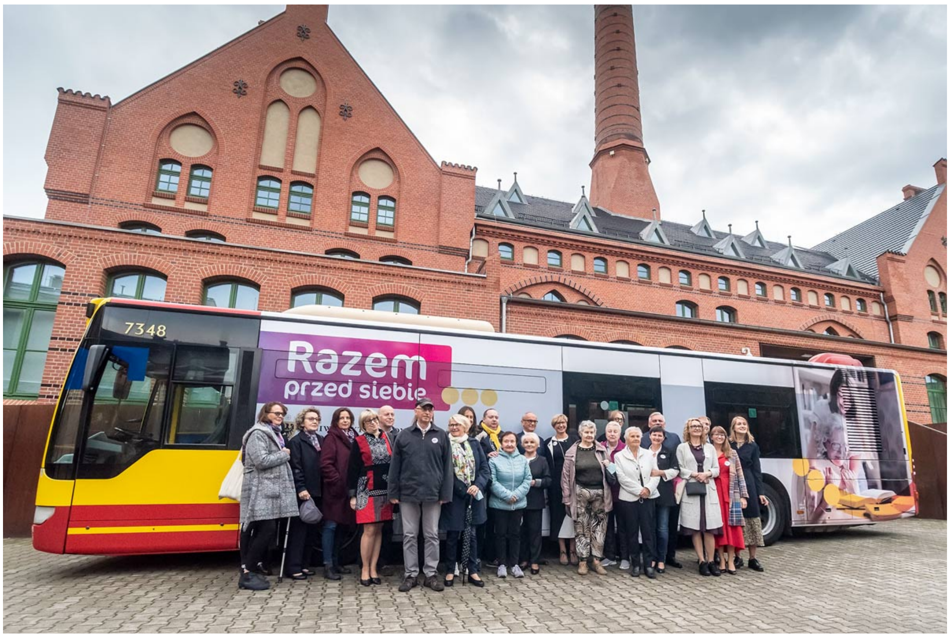

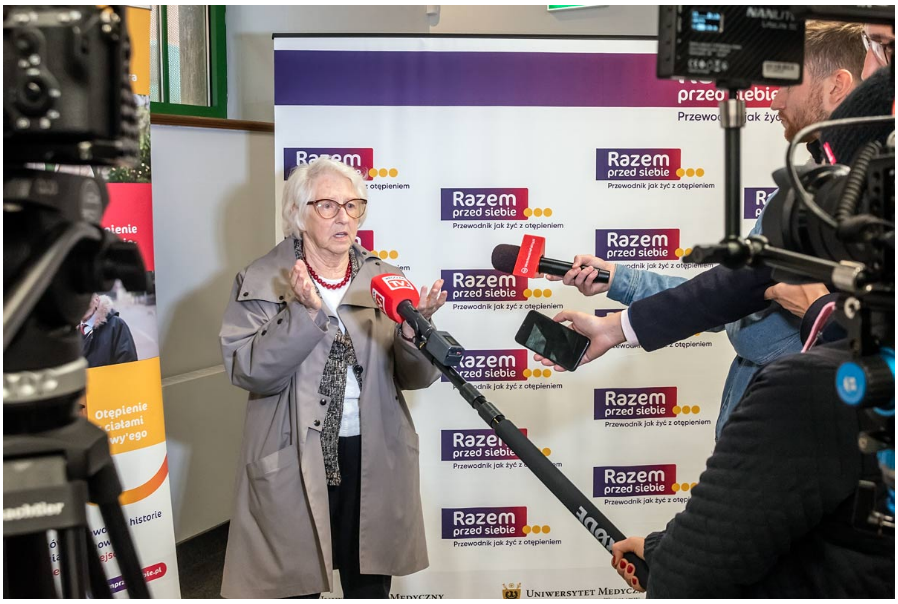

World Alzheimer's Day – 21st September 2021

Wrocław Sport Stadium lit up in the official colors of the Alzheimer's disease

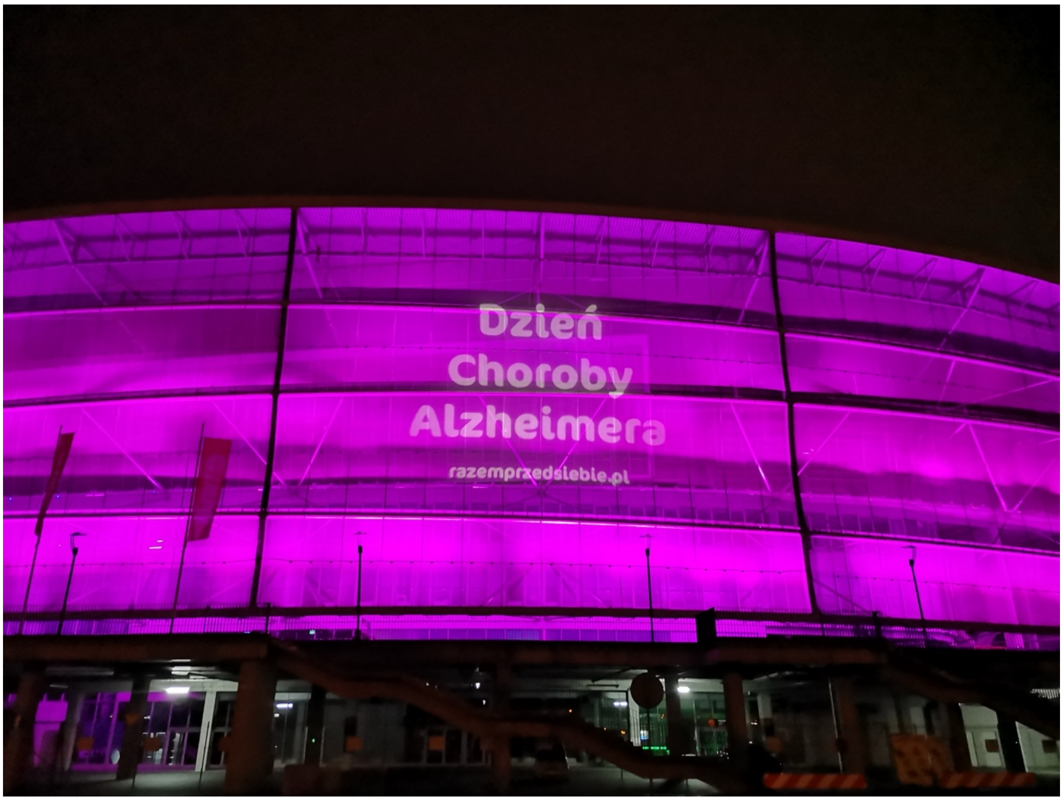

Wrocław Municipal Council Building lit up in the official colors of the Alzheimer's disease

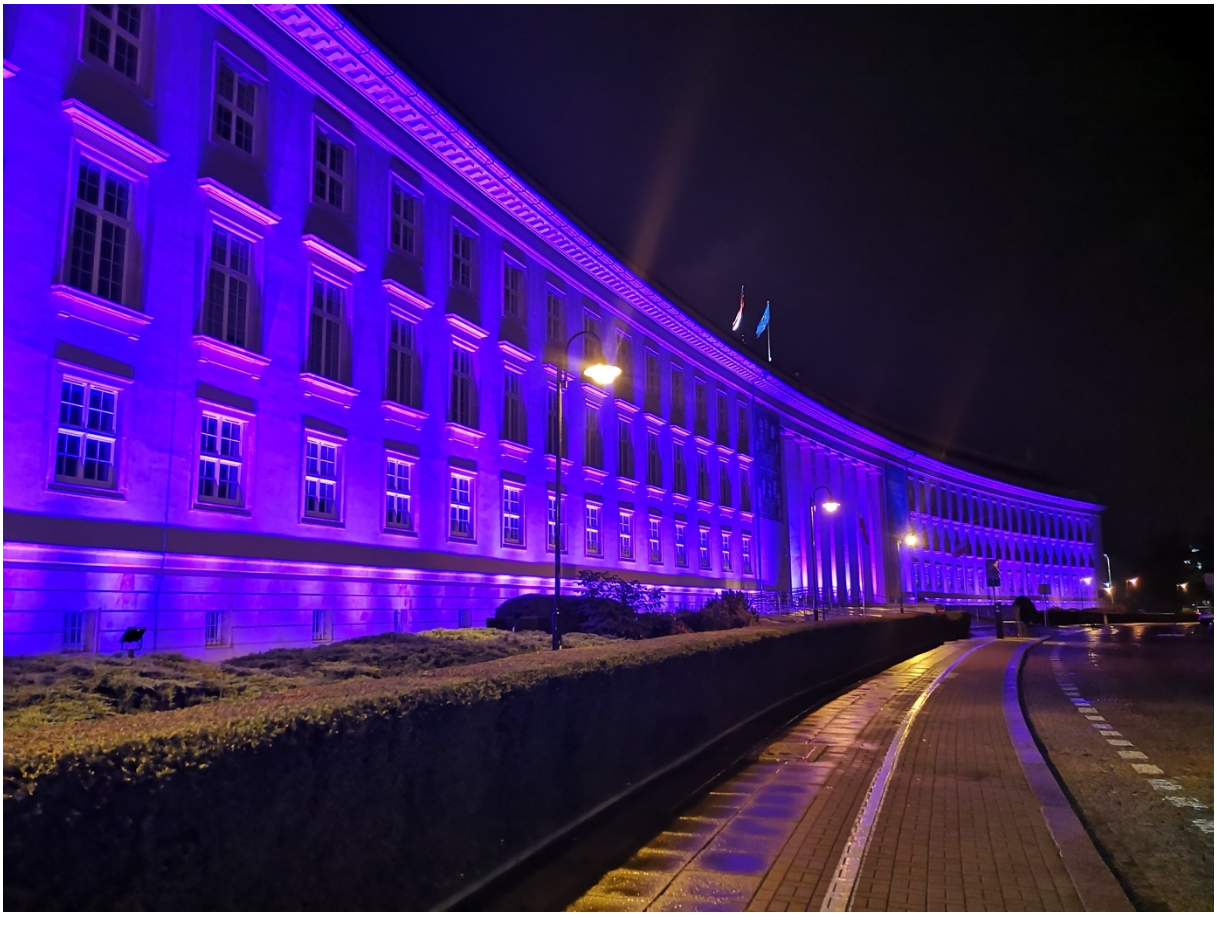

Archipelago of Kindness picnic at the Meeting Centre for people with dementia – 30th September 2021

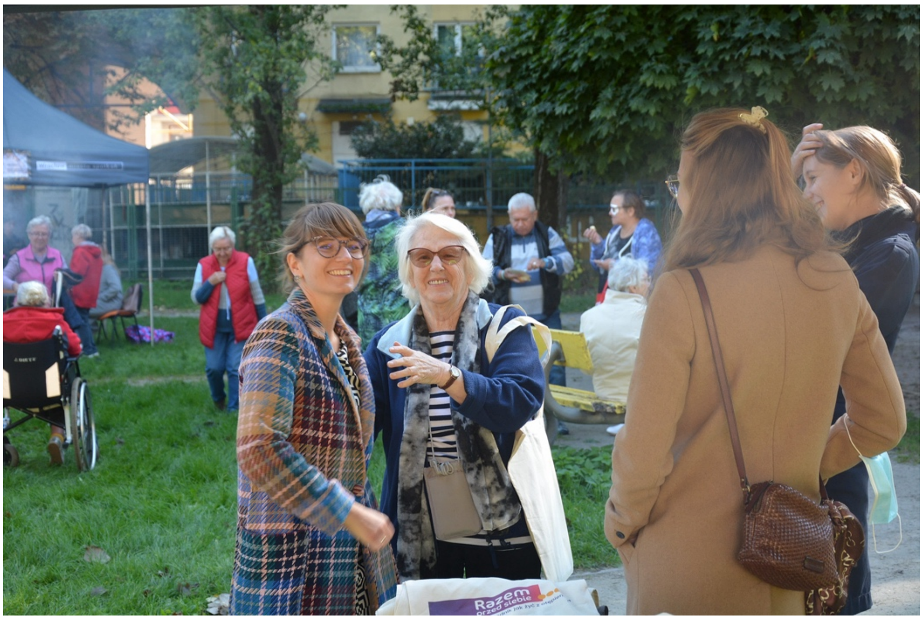

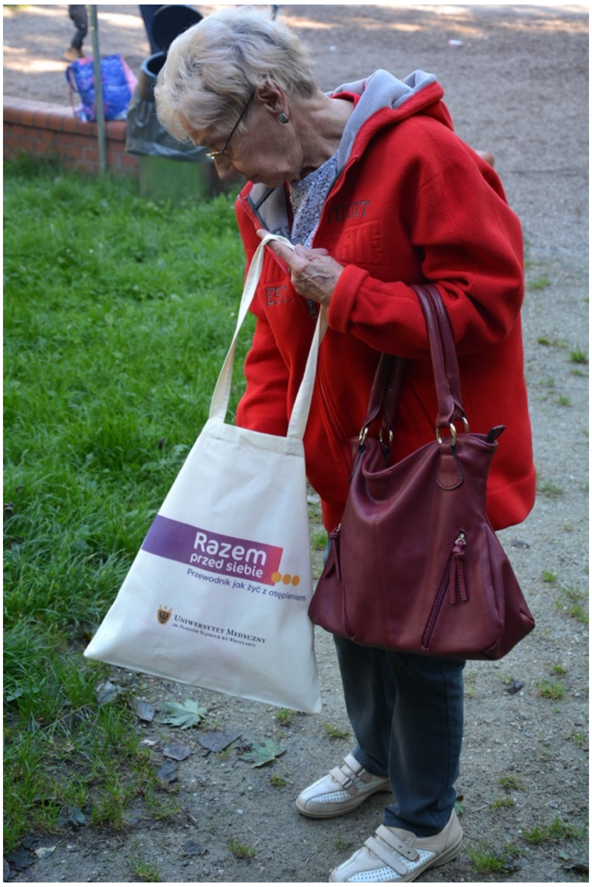

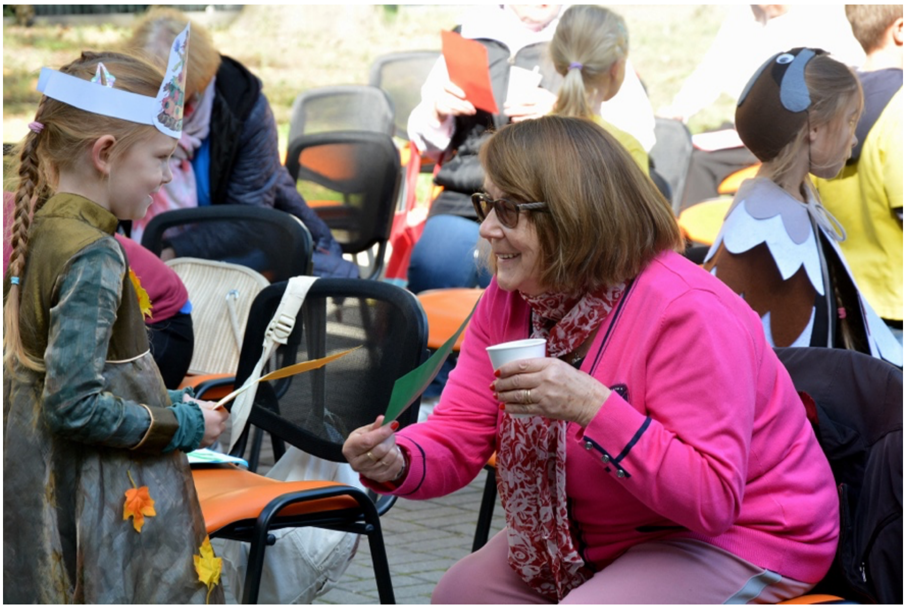

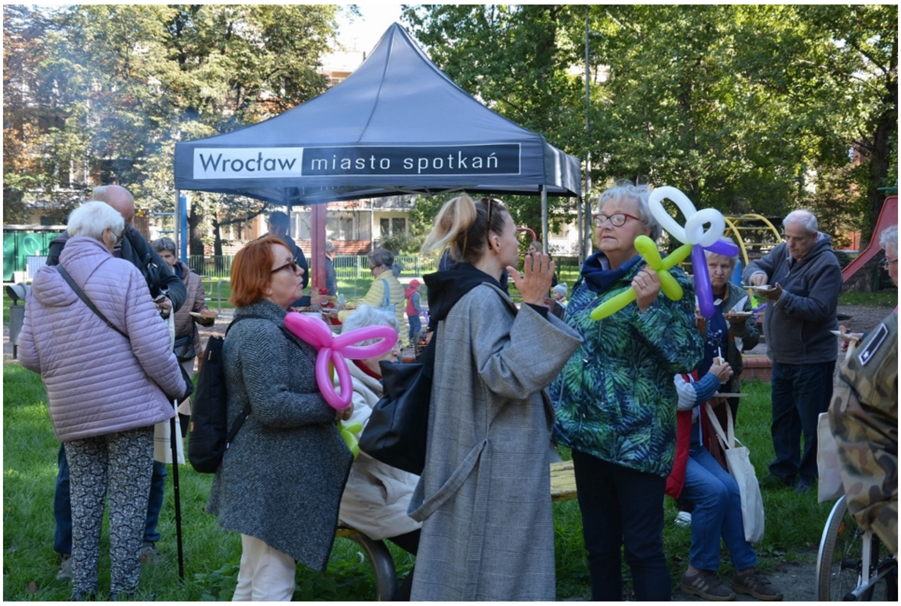

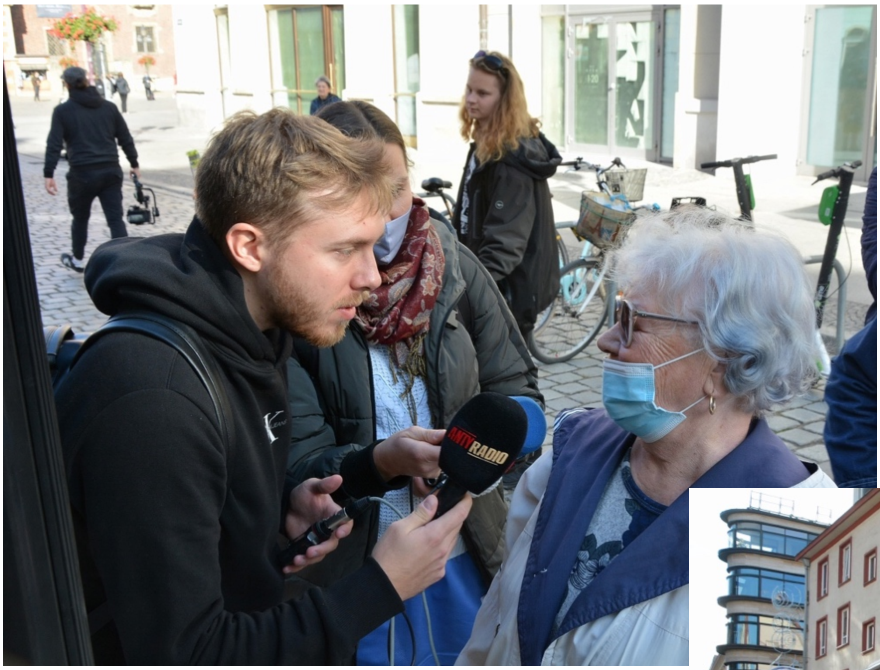

Campaign bus as a mobile diagnostic point
in the Wrocław City Centre
–
1st October 2021

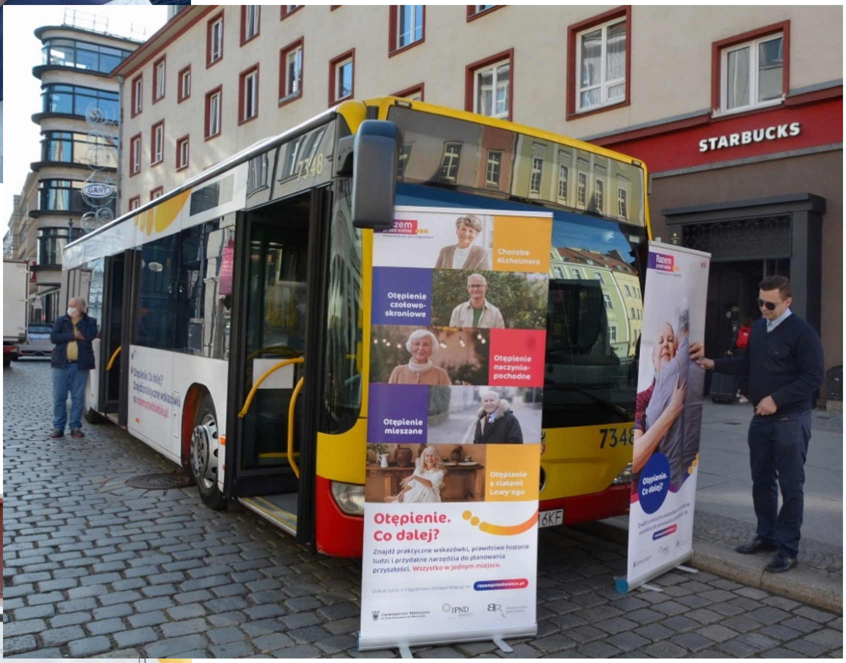

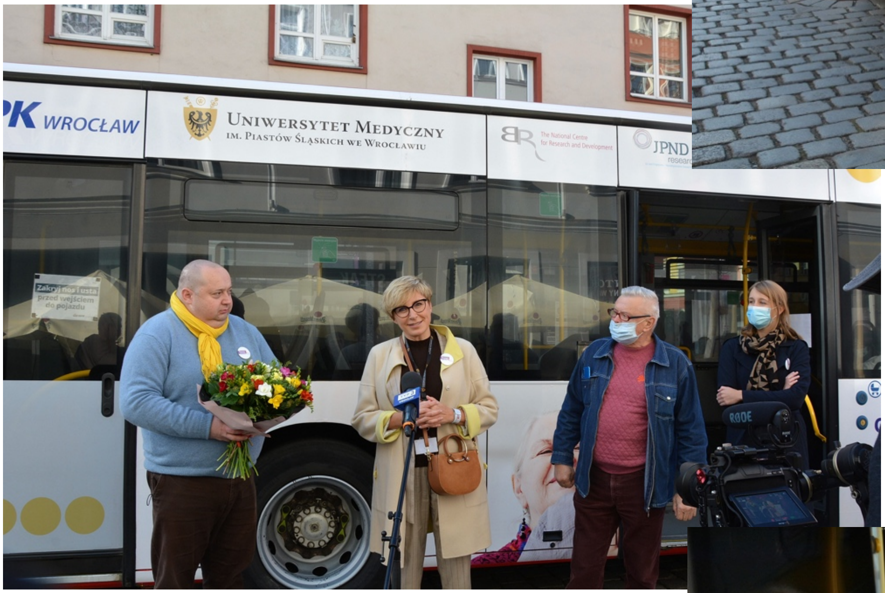

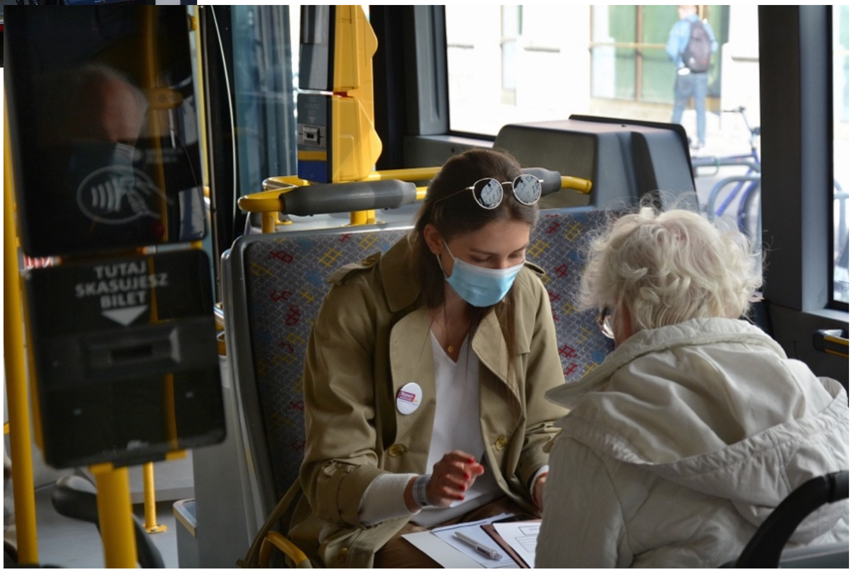

Vernissage, Art Collection created by an Artist living with a young-onset dementia displayed in the Wrocław City Centre and at the University Hospital
9th October 2021 – 25th October 2021

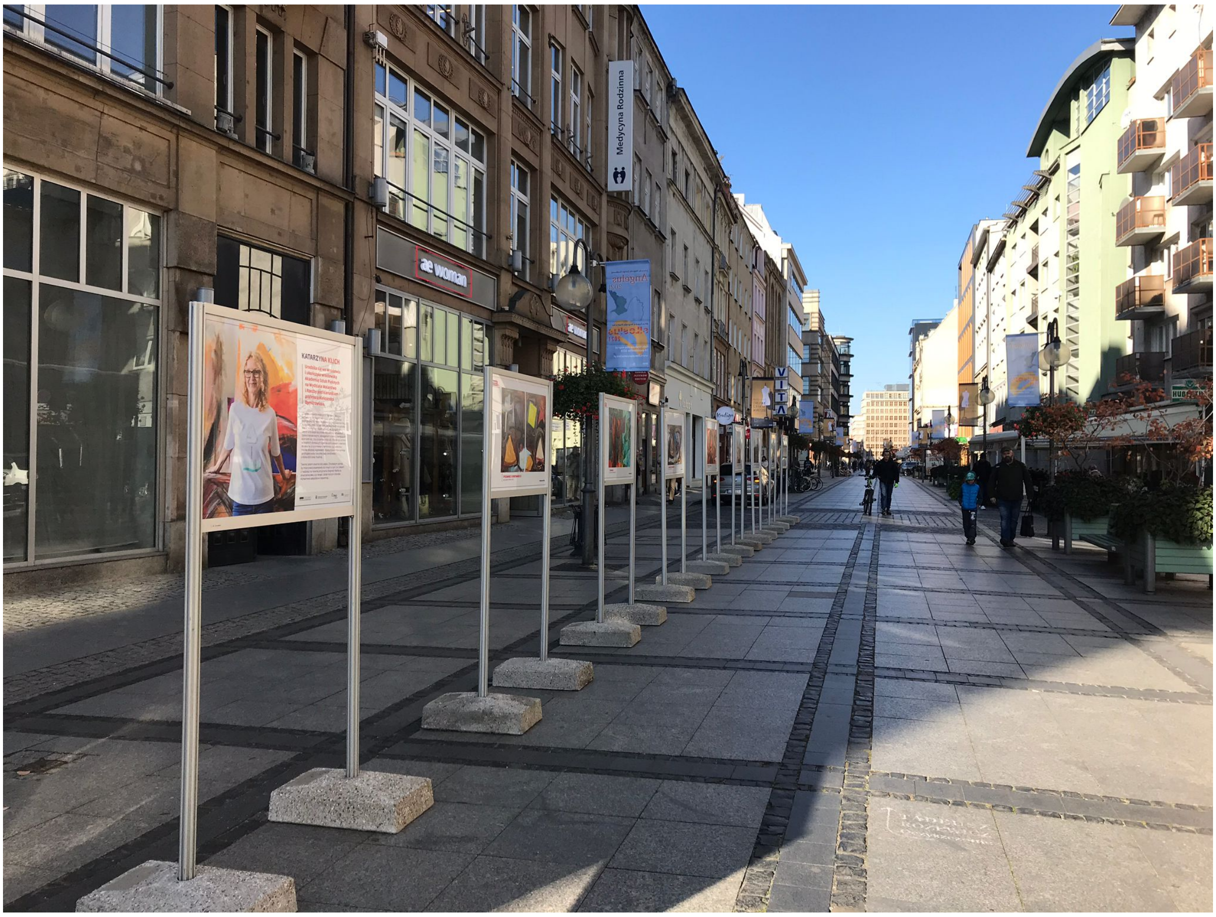

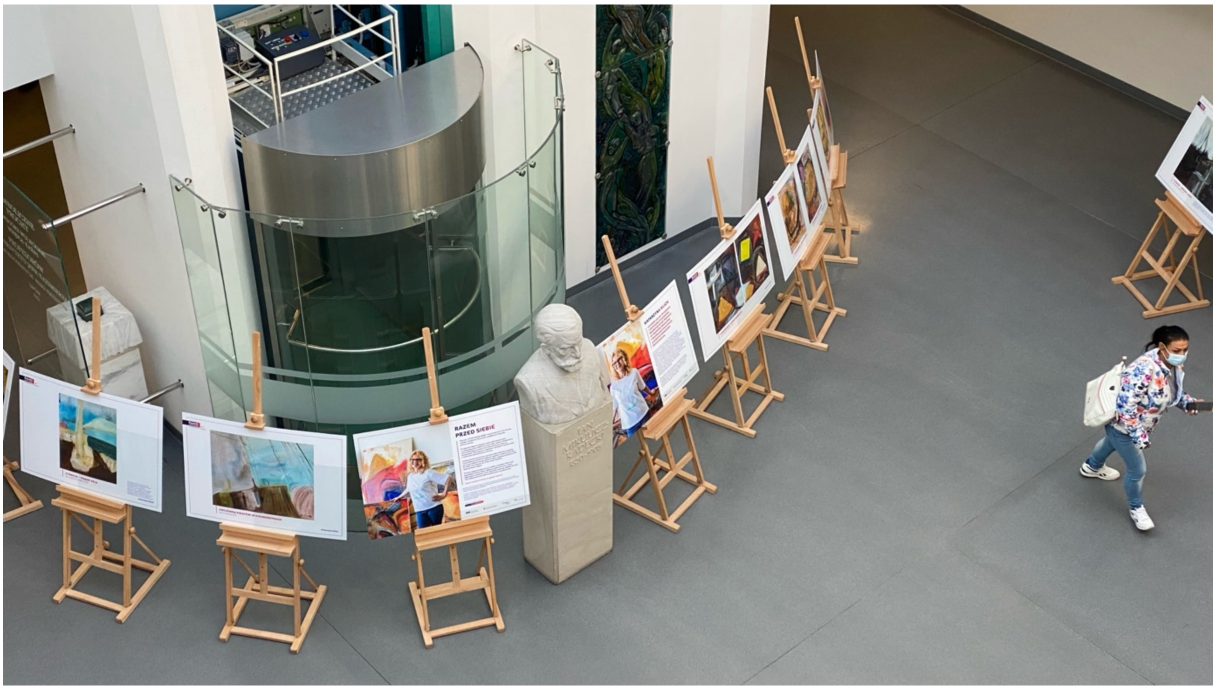

Concert titled *A song is a best remedy* as the official ending of the *Razem przed siebie* campaign – 1st February 2022

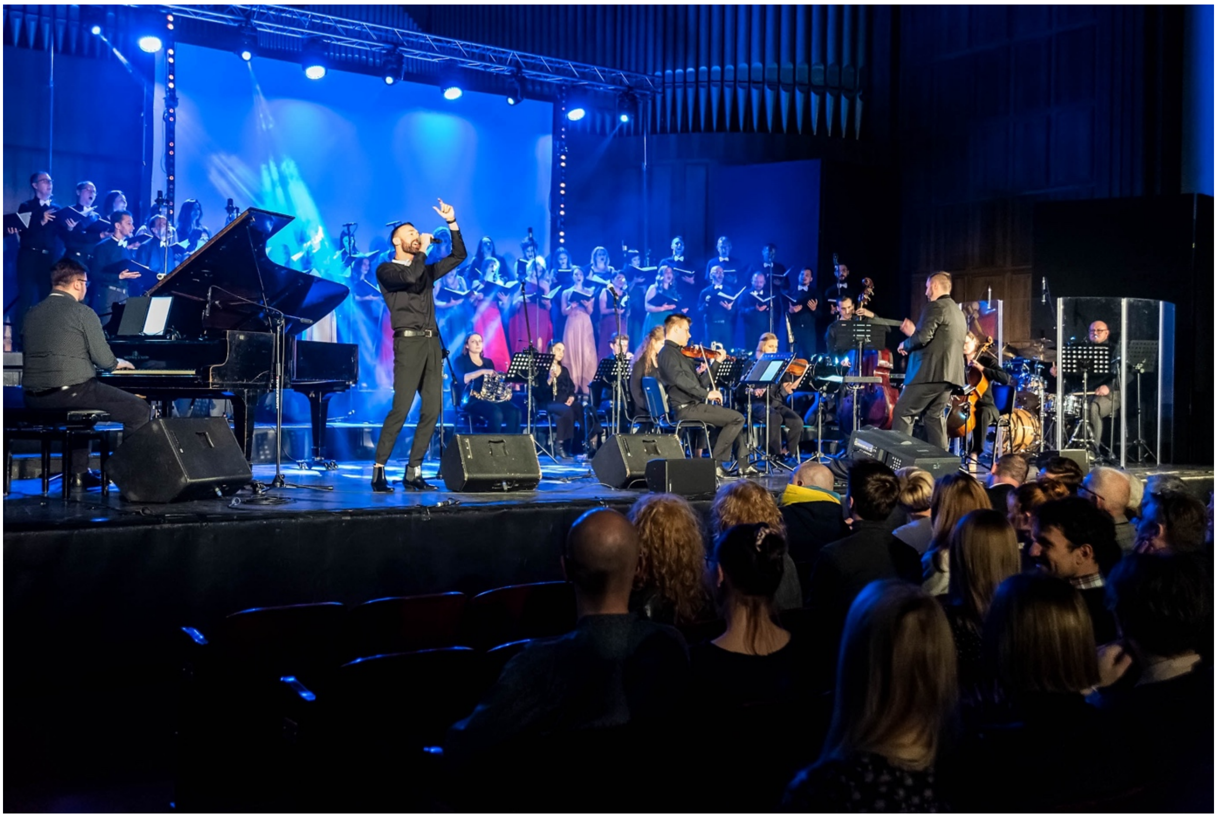

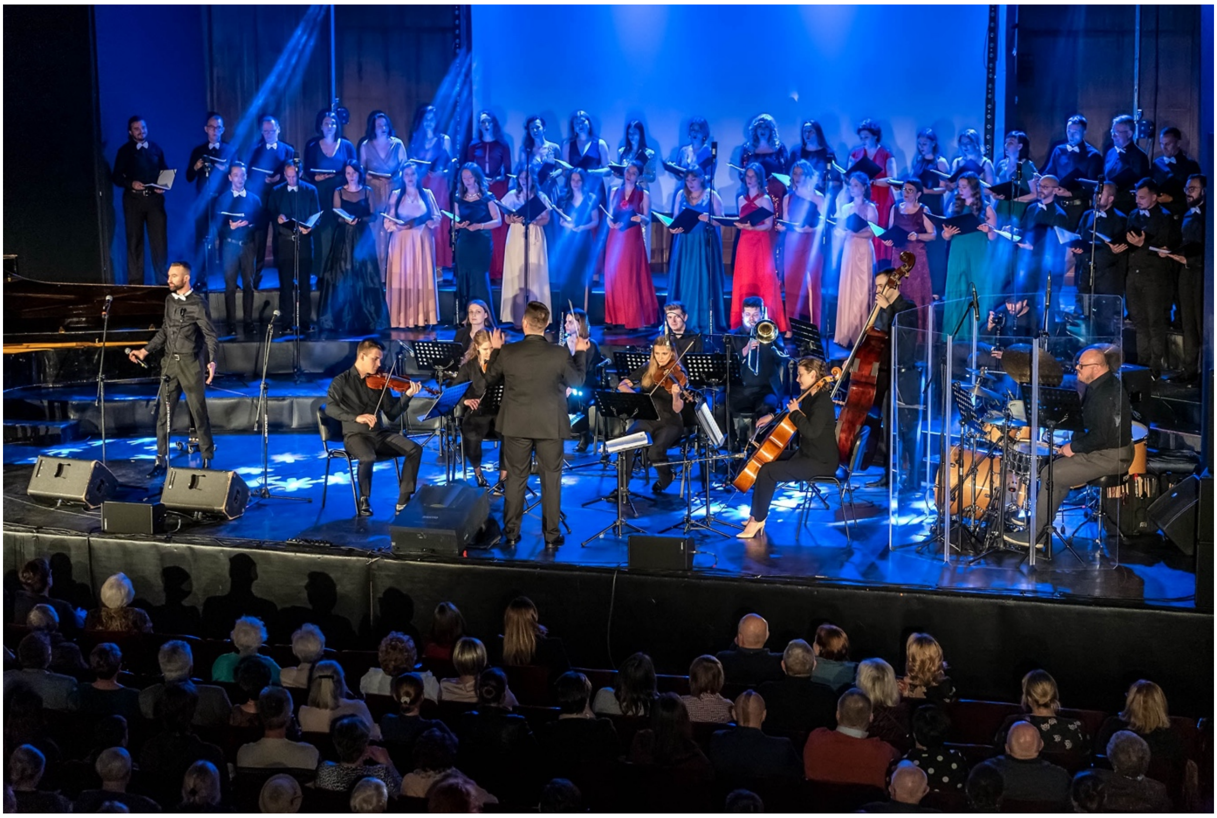

The author of the photos is Tomasz Walów.
